# Cardiovascular risk assessment and risk factor control in patients with hypertension in Europe: the SNAPSHOT study

**DOI:** 10.1093/ehjqcco/qcag002

**Published:** 2026-01-19

**Authors:** Dragos Vinereanu, Miguel Camafort, Anastase Dzudie, Branislava Ivanovic, José María Mostaza, Ivan Pećin, Arman Postadzhiyan, Tamaz Shaburishvili, Julien Magne

**Affiliations:** Cardiology and Cardiovascular Surgery Department, University of Medicine and Pharmacy Carol Davila, Bucharest 020021, Romania; Department of Cardiology, University and Emergency Hospital, Splaiul Independentei 169, Bucharest 050098, Romania; Hospital Clínic of Barcelona, University of Barcelona, Barcelona 08036, Spain; Centro de Investigación Biomédica en Red-Fisiopatología de la Obesidad y Nutrición (CIBEROBN), ISCIII, Madrid 28029, Spain; Cardiology & Cardiac Pacing Unit, Douala General Hospital, PO Box 4856, Douala, Cameroon; Clinical Research Education, Networking and Consultancy, PO Box 3787, Yaounde, Cameroon; Cardiology Clinic, University Clinical Centre of Serbia, Belgrade 11000, Serbia; Lipid and Vascular Risk Unit, Hospital La Paz-Carlos III, Madrid 28029, Spain; Faculty of Medicine of the Universidad Autonoma de Madrid, Madrid 28040, Spain; School of Medicine, University of Zagreb, Zagreb 10000, Croatia; Department of Internal Medicine, University Hospital Center Zagreb, Zagreb 10000, Croatia; Department of General Medicine, Medical University of Sofia, Sofia 1431, Bulgaria; Department of Cardiology, St Anna University Hospital, Sofia 1709, Bulgaria; Tbilisi Heart and Vascular Clinic, Ilia State University of Georgia, Tbilisi, Georgia; Center of Clinical and Research Data, Epimact Inserm 1094, IRD U270, University Hospital of Limoges, CHU de Limoges, Limoges 87000, France

**Keywords:** Blood pressure control, Cardiovascular risk, Dyslipidaemia, Epidemiological study, Hypertension, LDL-C control

## Abstract

**Aims:**

Underestimated cardiovascular (CV) risk may lead to inadequate control of blood pressure (BP), LDL cholesterol, and glycated haemoglobin. This study investigated CV risk assessment and BP, LDL cholesterol, and glycated haemoglobin control among patients with hypertension in routine clinical practice.

**Methods and results:**

In the observational, cross-sectional, epidemiological SNAPSHOT study (conducted in Bulgaria, Croatia, Georgia, Romania, Serbia, and Spain), CV risk was assessed in adults with hypertension according to the physician clinical practices, guidelines, and the risk assessment models valid when the study was performed (SCORE1 and SCORE2/SCORE2-OP). Blood pressure, LDL cholesterol, and glycated haemoglobin control rates were also assessed. Of 9307 patients (aged 65.8 ± 10.5 years, 43.1% male), most (91.3%) had ≥1 additional CV risk factor; 7610 (81.8%) had dyslipidaemia and 3097 (33.3%) had type 2 diabetes (T2D). Compared with guideline recommendations, assessment of patient CV risk by physicians, relative to the risk obtained using SCORE1 and SCORE2/SCORE2-OP, was accurate in only 38.0 and 26.7% of patients, respectively, and was underestimated in 54.3 and 71.8% of patients. Control rates of BP, LDL cholesterol, and glycated haemoglobin were suboptimal [<25%, <12% (in those with comorbid dyslipidaemia), and <50% (in those with comorbid T2D), respectively].

**Conclusion:**

Physicians from six European countries tended to overestimate control rates of BP and LDL cholesterol, while underestimating CV risk in their patients with hypertension. Overall, BP, LDL cholesterol, and glycated haemoglobin control rates were low. Better implementation of clinical guideline recommendations is needed.

## Graphical Abstract

**Graphical Abstract:**
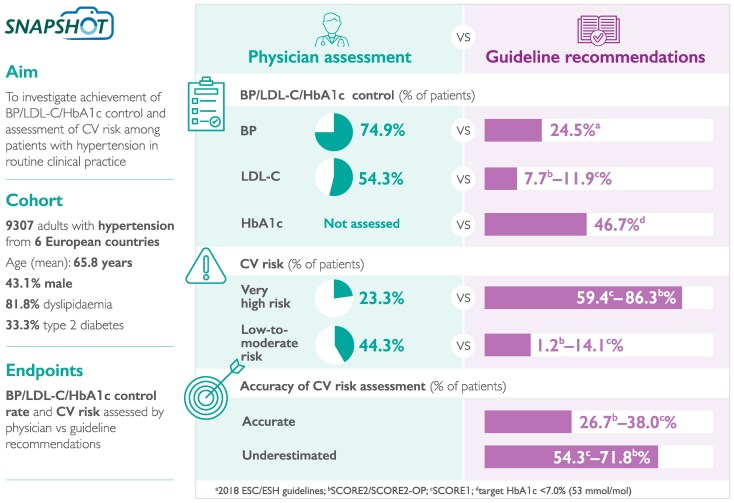

Key Learning Points What is already known:

- Current European guidelines provide recommendations for cardiovascular (CV) risk assessment in patients with hypertension, who often have comorbid dyslipidaemia and type 2 diabetes.
- Underestimation of CV risk by physicians may lead to therapeutic inertia and suboptimal control of blood pressure (BP), LDL-cholesterol (LDL-C), and glycated haemoglobin (HbA1c).
- There is an unmet need to understand physician perception of CV risk and the lack of BP, LDL-C, and HbA1c control in patients with hypertension.

What this study adds:

- The observational, cross-sectional SNAPSHOT study of physicians from six European countries found that CV risk tended to be underestimated among patients with hypertension in routine clinical practice, while control of BP and LDL-C tended to be overestimated.
- The proportion of patients achieving BP, LDL-C, and HbA1c targets was low, indicating concern for therapeutic inertia in these patients.
- These findings highlight the need for improvements in the assessment and management of CV risk in patients with hypertension.

## Introduction

Hypertension is a leading preventable risk factor for cardiovascular (CV) disease; an estimated 8–10 million deaths per year can be attributed to high blood pressure (BP).^1^ Globally, the number of people with hypertension doubled between 1990 and 2019, with hypertension estimated to affect almost 1.3 billion people in 2019.^1^ However, the prevalence differs by country, age, and sex.^1–3^

In addition to hypertension, other risk factors for CV disease, such as dyslipidaemia and type 2 diabetes (T2D), often occur concurrently, and the overall CV risk is the result of an interplay of these risk factors.^4–6^ Current European guidelines propose specific CV risk assessment, treatment recommendations, and BP targets for patients with hypertension with or without dyslipidaemia and/or T2D.^7–10^ Despite these clear recommendations, adequate control of BP, LDL cholesterol (LDL-C), and glycated haemoglobin (HbA1c) is insufficient, and remain challenging in patients with multiple comorbidities.^11–16^ Furthermore, the number of comorbidities increases with age, requiring complex drug regimens, which may lead to low adherence and poorer control of CV risk.^13^ An additional factor that may contribute to suboptimal CV risk control in patients is physician underestimation of patient CV risk,^17–19^ which may lead to therapeutic inertia. Patient-related factors that might lead to poor CV risk control have been frequently investigated, but the contribution of physicians to less-than-ideal target achievement (i.e. better CV risk control) is much less studied.^17^

Therefore, there is an unmet need to understand physician perception of CV risk and the lack of BP, LDL-C, and HbA1c control in patients with hypertension. As such, we aimed to investigate CV risk perception vs. assessment, and the consequent achievement of BP control in patients with hypertension, as well as LDL-C and HbA1c control in hypertensive patients with comorbid dyslipidaemia and T2D, respectively.

## Methods

### Design

SNAPSHOT was an observational, cross-sectional, epidemiological study conducted at 447 centres in 6 European countries: Bulgaria (134 centres), Croatia (100), Georgia (30), Romania (133), Serbia (22), and Spain (28). Eligible centres were any hospital, outpatient clinic, or primary care facility that treated patients with hypertension. Participating physicians (*n* = 513) were asked to enrol consecutive eligible patients within a defined period (2–4 weeks according to the study protocol), between April 2020 and September 2022. A summary of the clinical centre types for the participating physicians is provided in Supplementary material online, *Table S1*.

The study was conducted in accordance with the Declaration of Helsinki (2013 revision), applicable regulatory requirements in each country, and relevant national guidelines for the protection of patient confidentiality, including the General Data Protection Regulation in Europe. The study protocol was approved by the relevant Ethics Committees and/or Regulatory Authorities according to each country’s requirements. Oral and/or written informed consent was obtained from each participant in the local language, and in accordance with local research ethics requirements.

### Patients

All patients aged ≥18 years (or legal age) attending the clinic for a routine visit, and with a diagnosis of sustained hypertension [defined as systolic BP (SBP) ≥ 140 mmHg and/or diastolic BP (DBP) ≥ 90 mmHg, or already receiving antihypertensive therapy] were included in the SNAPSHOT study, provided they give their informed consent. In four countries (Croatia, Romania, Serbia, and Spain), patients also had to have a diagnosis of dyslipidaemia [defined as LDL-C ≥ 116 mg/dL (≥3.0 mmol/L) or already receiving lipid-lowering drugs (LLDs)]. Patients who were unwilling or unable to give consent were excluded.

### Assessments

Treatment and care were maintained according to clinical practice, and no specific investigations or therapies were recommended as part of this study. The following data were collected by the investigator during a single visit (routine visit at site), entered into an electronic case report form, and de-identified prior to analysis: (i) demographic information; (ii) risk factors, comorbidities, and treatments; (iii) BP recorded as per the European Society of Cardiology and European Society of Hypertension (ESC/ESH) 2018 guidelines (three measurements, 1–2 min apart, with the average of the last two recorded^20^); (iv) heart rate seated and at rest; (v) presence of comorbid dyslipidaemia [in these patients, investigators recorded the most recent results for total cholesterol, HDL cholesterol (HDL-C), LDL-C, and triglycerides from patient’s records within the last year]; (vi) presence of comorbid T2D [HbA1c > 6.5% (>48 mmol/mol) or receiving treatment with glucose-lowering drugs (GLDs); investigators recorded the most recent results for HbA1c, fasting glucose, GLDs, and complications from patient’s records within the last year].

Based on the collected data, CV risk for each patient was assessed according to:

- The physician-assessed risk category was recorded in the electronic case report form by the investigator according to their medical judgement (risk categories defined as low, moderate, high, or very high);
- The Systematic COronary Risk Evaluation chart 1 (SCORE1) risk prediction algorithm, proposed by Piepoli and colleagues and revised in the 2019 ESC/European Atherosclerosis Society (EAS) guidelines (risk categories defined as low, moderate, high, or very high; Supplementary material online, *Table S2*);^10,21^ guideline recommendations;^20^ and 2021 SCORE2 (for adults aged 40–69 years^22^) or 2021 SCORE2-OP (for adults aged ≥70 years^23^) risk prediction algorithms (risk categories defined as low-to-moderate, high, or very high; Supplementary material online, *Table S3*).

For ‘apparently’ healthy patients, the SCORE2/SCORE2-OP guidelines restrict the calculation of CV risk based on specific age groups (40–79 years), SBP values (110–179 mmHg), and non-HDL-C values (116–268 mg/dL). Therefore, to calculate CV risk for patients with out-of-range parameters, the values of these parameters were replaced by the nearest borderline values from the SCORE2/SCORE2-OP chart. For ‘apparently’ healthy patients with missing non-HDL-C values, it was possible to calculate the CV risk category for some patients according to SCORE2/SCORE2-OP criteria using all other parameters (age, sex, SBP, and smoking status) to identify patients whose SCORE2/SCORE2-OP CV risk category remained consistent across all non-HDL-C values.

Blood pressure control rates were evaluated in all patients using the perception of the physician according to their clinical practice (physician assessment) and according to achievement of BP targets outlined in the 2018 ESC/ESH guidelines (see Supplementary material online, *Table S4*).^20^ LDL-C control rates were evaluated in patients with a diagnosis of dyslipidaemia using the perception of the physician according to their clinical practice (physician assessment) and achievement of SCORE1 and/or SCORE2/SCORE2-OP LDL-C targets (see Supplementary material online, *Table S5*).^21-23^ HbA1c control rates were evaluated in patients with a diagnosis of T2D according to the achievement of an HbA1c < 7% (<53 mmol/mol), per the American Diabetes Association 2020 glycaemic targets.^24^ Blood pressure, LDL-C, and HbA1c control rates were also assessed in the following subgroups: CV risk category (physician’s assessment, SCORE1, SCORE2/SCORE2-OP), accuracy of physician’s CV risk assessment vs. SCORE1 and SCORE2/SCORE2-OP, age, sex, body mass index (BMI), and the presence of comorbidities [dyslipidaemia, T2D, coronary artery disease (CAD)]. For the purposes of this study, CAD was defined as a history of angina, myocardial infarction, acute coronary syndrome, or coronary or other arterial revascularization.

### Statistical analysis

The sample size required per country was calculated based on the estimated prevalence of patients with controlled hypertension in the country, with a risk *α* of 5% and a margin of error of 4%. Since the BP control rate varied between 20 and 50% per country, the minimum number of patients to be included was between 400 and 600, depending on the country. To be included in the analyses for this study, patients had to have hypertension and complete BP (SBP and DBP) data. Analyses were descriptive, with categorical data described using numbers, proportion of patients, and 95% confidence intervals (CI; calculated using the Wald method), and continuous data described using means and standard deviations (SD), as appropriate. When calculating the control rates of BP, LDL-C, and HbA1c, patients with missing data were considered as having ‘inconclusive’ control and were excluded from the denominator value. The combination of two or more factors was only considered ‘controlled’ when all factors were controlled; when one or more of these factors were ‘uncontrolled’, the status of the combination was considered ‘uncontrolled’, regardless of the status of the other factors; if one or two factors of a combination were ‘controlled’ but the second or third factor was missing (or ‘inconclusive’), these patients were excluded from the analysis. The *χ*^2^ tests were conducted *post hoc* to compare the following: CV risk between assessment methods (based on contingency tables); prevalence estimates of patients with controlled BP, LDL-C, and HbA1c between calculated CV risk categories (SCORE1 and SCORE2/SCORE2-OP); the level of physician’s accuracy of assessment of CV risk vs. calculated CV risk according to SCORE1 and SCORE2/SCORE2-OP risk algorithms; and BP, LDL-C, and HbA1c control rates in various subgroups (age, sex, BMI, and comorbidities). A *P*-value of <0.05 was considered to indicate statistical significance. All statistical analyses were conducted using SAS version 9.4 or higher.

## Results

### Patients

Overall, 9466 patients with hypertension were included in the study, 9307 had data suitable for analysis, including 3260 patients (35.0%) from Bulgaria, 1296 (13.9%) from Croatia, 585 (6.3%) from Georgia, 2522 (27.1%) from Romania, 1180 (12.7%) from Serbia, and 464 (5.0%) from Spain. One hundred and fifty-nine patients were excluded from the analysis, including 23 patients who did not meet the inclusion criteria and 136 patients who had missing or aberrant values. The study population (mean ± SD age 65.8 ± 10.5 years) included a higher proportion of females than males (56.9 vs. 43.1%; *Table 1*).

**Table 1:** Demographic and clinical characteristics of patients in the SNAPSHOT study

	Patients (*n* = 9307)
Sex, *n* (%)	*n* = 9277
Male	3996 (43.1)
Age, years, mean ± SD	65.8 ± 10.5
≥65 years, *n* (%)	5506 (59.2)
Current smoker, *n* (%)	1594 (17.2)^a^
Race, *n* (%)	*n* = 9194
White	8748 (95.2)
Black or African	13 (0.1)
Asian	5 (<0.1)
Other	428 (4.7)
Ethnicity, *n* (%)	*n* = 9208
Hispanic or Latino	439 (4.8)
Not Hispanic or Latino	8769 (95.2)
Speciality of physician, *n* (%)	
General practitioner	6628 (71.2)
Cardiology	1584 (17.0)
Endocrinology	625 (6.7)
Internal medicine	420 (4.5)
Neurology	50 (0.5)
Institute type, *n* (%)	*n* = 9290
Outpatient clinic	6791 (73.1)
Hospital	1473 (15.9)
Primary care facility	1026 (11.0)
Location of institute, *n* (%)	*n* = 9290
Urban	7793 (83.9)
Rural	1497 (16.1)
BMI, *n* (%)	*n* = 9283
<18.5 kg/m^2^	27 (0.3)
18.5–25 kg/m^2^	1673 (18.0)
25–30 kg/m^2^	3912 (42.1)
≥30 kg/m^2^	3671 (39.6)
BP, mmHg, mean ± SD	*n* = 9307
SBP	139.3 ± 17.3
DBP	82.3 ± 10.8
Time since hypertension diagnosis, years, mean ± SD	11.7 ± 12.6^b^
Number of additional risk factors,^c^ *n* (%)	
0	808 (8.7)
1	3555 (38.2)
2	3700 (39.8)
3	1164 (12.5)
4	80 (0.9)
Number of additional comorbidities, *n* (%)	*n* = 9244
0	2776 (30.0)
1	2618 (28.3)
2	1734 (18.8)
3	1054 (11.4)
4	570 (6.2)
≥5	492 (5.3)
Comorbidities/target organ damage, *n* (%)	
Dyslipidaemia	7610 (81.8)^d^
Diabetes mellitus	3159 (34.0)^e^
T2D	3097 (33.3)^e^
CAD	2894 (31.2)^f^
Angina	2025 (21.8)^f^
Elective coronary or other arterial revascularization	777 (8.4)^f^
MI	741 (8.0)^f^
ACS	367 (3.9)^f^
CKD	870 (9.4)^e^
LDL-C, mg/dL, mean ± SD	119.7 ± 46.2^g^
HbA1c, %, mean ± SD	7.1 ± 1.3^h^

ACS, acute coronary syndrome; CKD, chronic kidney disease; MI, myocardial infarction.

^a^ *n* = 9294.

^b^ *n* = 9270.

^c^Risk factors in addition to hypertension were male sex, age ≥65 years, BMI ≥30 kg/m^2^, and smoking.

^d^ *n* = 9302.

^e^ *n* = 9301.

^f^ *n* = 9273.

^g^In patients with dyslipidaemia (*n* = 7458).

^h^In patients with T2D (*n* = 2447).

Based on BMI, >80% of the patient population were either overweight (25–30 kg/m^2^; 42.1%) or obese (≥30 kg/m^2^; 39.6%). Most patients (91.3%) had at least one CV risk factor in addition to hypertension (i.e. male sex, age ≥65 years, BMI ≥30 kg/m^2^ and/or smoking), while 70.0% had one or more additional comorbidities. The majority of patients had dyslipidaemia (81.8%), 34.0% had diabetes mellitus (33.3% with T2D), 31.2% had CAD, and 9.4% had chronic kidney disease.

### Cardiovascular risk assessment

**Figure 1:**
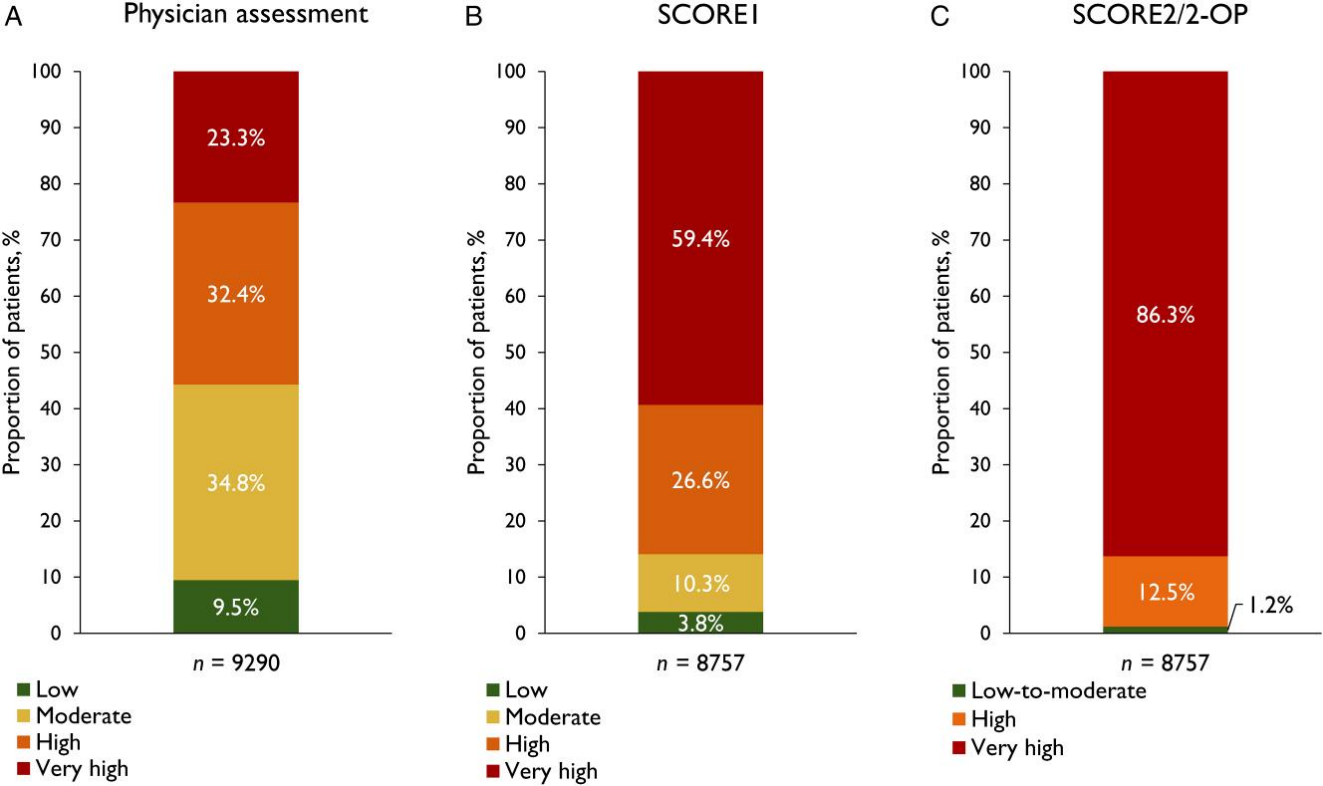
Cardiovascular risk according to (*A*) physician assessment, (*B*) guideline recommendations and SCORE1 prediction algorithm, or (*C*) guideline recommendations and SCORE2/SCORE2-OP prediction algorithm. OP, older persons.

According to guideline recommendations and the SCORE1 or SCORE2/SCORE2-OP risk prediction algorithms, most patients had very high CV risk (59.4% using SCORE1 and 86.3% using SCORE2/SCORE2-OP), whereas only 23.3% of patients had very high CV risk when assessed by physicians (*Figure 1A–C*).

**Figure 2:**
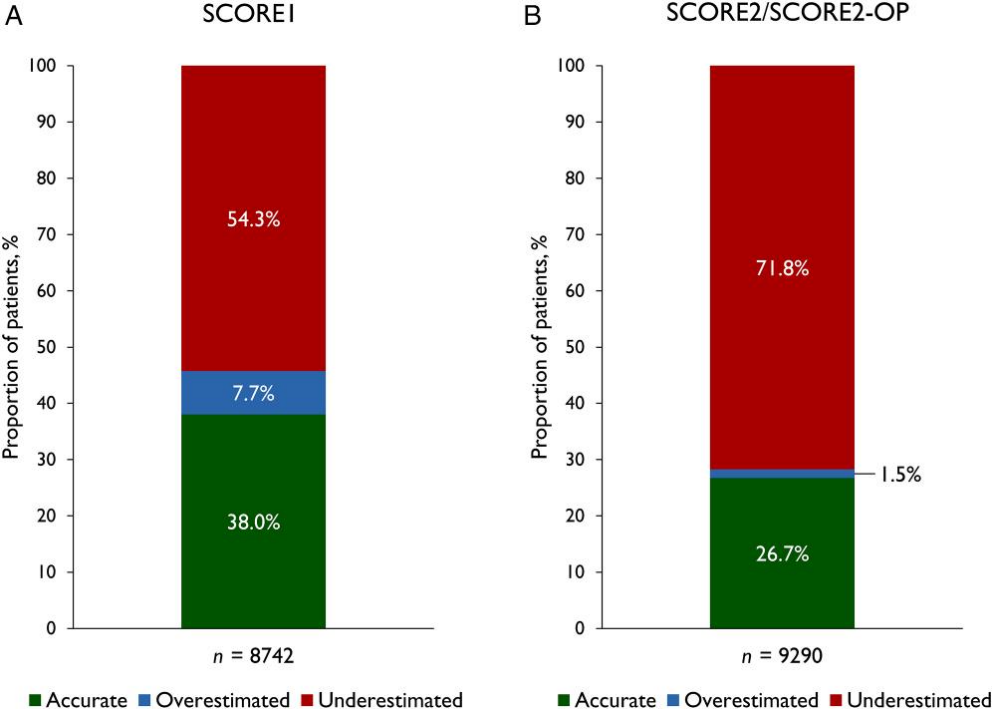
The accuracy of cardiovascular risk assessment by physicians relative to the (*A*) guideline recommendations and SCORE1 prediction algorithm or (*B*) guideline recommendations and SCORE2/SCORE2-OP prediction algorithm. OP, older persons.

Risk assessment categories were significantly different between physicians and guideline recommendations, using either SCORE1 (*P* < 0.0001) or SCORE2/SCORE2-OP (*P* < 0.0001) prediction algorithms, and also between the two prediction algorithms (SCORE1 vs. SCORE2/SCORE2-OP; *P* < 0.0001). Physician assessment of CV risk was considered accurate in 38.0% of patients relative to the use of SCORE1, and in 26.7% of patients relative to the use of SCORE2/SCORE2-OP. Physicians underestimated the CV risk in over half of the patient population, specifically in 54.3% of patients relative to the use of SCORE1 (*Figure 2A*), and in 71.8% of patients relative to the use of SCORE2/SCORE2-OP (*Figure 2B*).

A summary of the CV risk assessment categories across the six European countries is provided in Supplementary material online, *Table S6*.

### Control rates

Overall, the proportion of patients achieving BP, LDL-C, or HbA1c control was low. Thus, physicians overestimated BP control, with 74.9% of patients achieving BP control according to physician assessment, whereas 48.2% of patients had BP control according to achievement of the BP target <140/90 mmHg, and 24.5% had BP control according to 2018 ESC/ESH guidelines (*Table 2*).

**Table 2:** Rates of control for BP, LDL-C, and HbA1c according to different definitions

	Control rate, *n*/*N*^a^ (%) [95% CI]
BP control	
Physician assessment	6863/9160 (74.9) [74.0–75.8]
BP <140/90 mmHg	4486/9307 (48.2) [47.2–49.2]
2018 ESC/ESH guidelines^b^	2275/9305 (24.5) [23.6–25.3]
LDL-C control	
Physician assessment	3639/6706 (54.3) [53.1–55.5]
SCORE1^c^	885/7455 (11.9) [11.1–12.6]
SCORE2/SCORE2-OP^d^	575/7458 (7.7) [7.1–8.3]
BP + LDL-C control	
Physician assessment	3178/7376 (43.1) [42.0–44.2]
Controlled according to 2018 ESC/ESH guidelines^b^ and SCORE1^b^	289/7589 (3.8) [3.4–4.2]
Controlled according to 2018 ESC/ESH guidelines^b^ and SCORE2/SCORE2-OP^d^	184/7575 (2.4) [2.1–2.8]
HbA1c control	
HbA1c < 7% (<53 mmol/mol)^e^	1143/2447 (46.7) [44.8–48.7]
BP + HbA1c control	
Controlled according to 2018 ESC/ESH guidelines^b^ and HbA1c < 7% (<53 mmol/mol)^e^	340/2959 (11.5) [10.3–12.6]
BP + LDL-C + HbA1c control	
Controlled according to 2018 ESC/ESH guidelines,^b^ SCORE1,^c^ and HbA1c < 7% (<53 mmol/mol)^e^	53/2736 (1.9) [1.4–2.5]
Controlled according to 2018 ESC/ESH guidelines,^b^ SCORE2/SCORE2-OP,^d^ and HbA1c < 7% (<53 mmol/mol)^e^	51/2736 (1.9) [1.4–2.4]

ADA, American Diabetes Association; CKD, chronic kidney disease; OP, older persons.

^a^Patients with missing data were considered as having ‘inconclusive’ control and were excluded from the denominator value when calculating the control rate. The combination of two or more factors was only considered ‘controlled’ when all factors were controlled; when one or more of these factors were ‘uncontrolled’, the status of the combination was considered ‘uncontrolled’, regardless of the status of the other factors; if one or two factors of a combination were ‘controlled’ but the second or third factor was missing (or ‘inconclusive’), these patients were excluded from the analysis.

^b^2018 ESC/ESH guidelines: in non-treated patients (not under antihypertensive medication): (i) for patients aged <80 years, BP control is defined as BP <140/90 mmHg; (ii) for patients aged ≥80 years, BP control is defined as BP <160/90 mmHg. In treated patients: (i) for patients aged <65 years without CKD, BP control is defined as SBP ≤130 mmHg and DBP <80 mmHg; (ii) for patients aged <65 years with CKD and patients aged ≥65 years, BP control is defined as BP <140/80 mmHg.^20^

^c^SCORE1: For patients with very high CV risk (calculated), LDL-C control is defined as LDL-C < 1.4 mmol/L (<55 mg/dL). For patients with high CV risk (calculated), LDL-C control is defined as LDL-C < 1.8 mmol/L (<70 mg/dL). For patients with moderate CV risk (calculated), LDL-C control is defined as LDL-C < 2.6 mmol/L (<100 mg/dL). For patients with low CV risk (calculated), LDL-C control is defined as LDL-C < 3 mmol/L (<116 mg/dL).^10^

^d^SCORE2/SCORE2-OP: For patients with very high CV risk (calculated), LDL-C control is defined as LDL-C < 1.4 mmol/L (<55 mg/dL). For patients with high CV risk (calculated), LDL-C control is defined as LDL-C < 1.8 mmol/L (<70 mg/dL). For patients with low-to-moderate CV risk (calculated), LDL-C control is defined as LDL-C < 2.6 mmol/L (<100 mg/dL).^22,23^

^e^HbA1c control rates were evaluated per the ADA 2020 glycaemic targets.^24^

The rate of LDL-C control was also overestimated by physicians, with 54.3% of patients with dyslipidaemia achieving LDL-C control based on physician assessment, whereas only 11.9 and 7.7% of patients achieved LDL-C control based on SCORE1 and SCORE2/SCORE2-OP criteria, respectively (*Table 2*). In addition, physicians overestimated the proportion of patients with combined control of BP and LDL-C (43.1%), whereas combined control of BP and LDL-C, defined by 2018 ESC/ESH guidelines and SCORE1 or SCORE2/SCORE2-OP criteria, was 3.8 and 2.4%, respectively.

Less than half of all patients with T2D (46.7%) had adequate glycaemic control [target HbA1c < 7.0% (<53 mmol/mol); *Table 2*]. The rate of combined control of BP and HbA1c was 11.5%, while the rate of combined control of BP, LDL-C, and HbA1c was 1.9% when SCORE1 criteria were used, and 1.9% when SCORE2/SCORE2-OP criteria were used.

A summary of the control rates across the different countries is provided in Supplementary material online, *Table S6*.

### Control rates according to cardiovascular risk

#### Calculated cardiovascular risk

**Figure 3:**
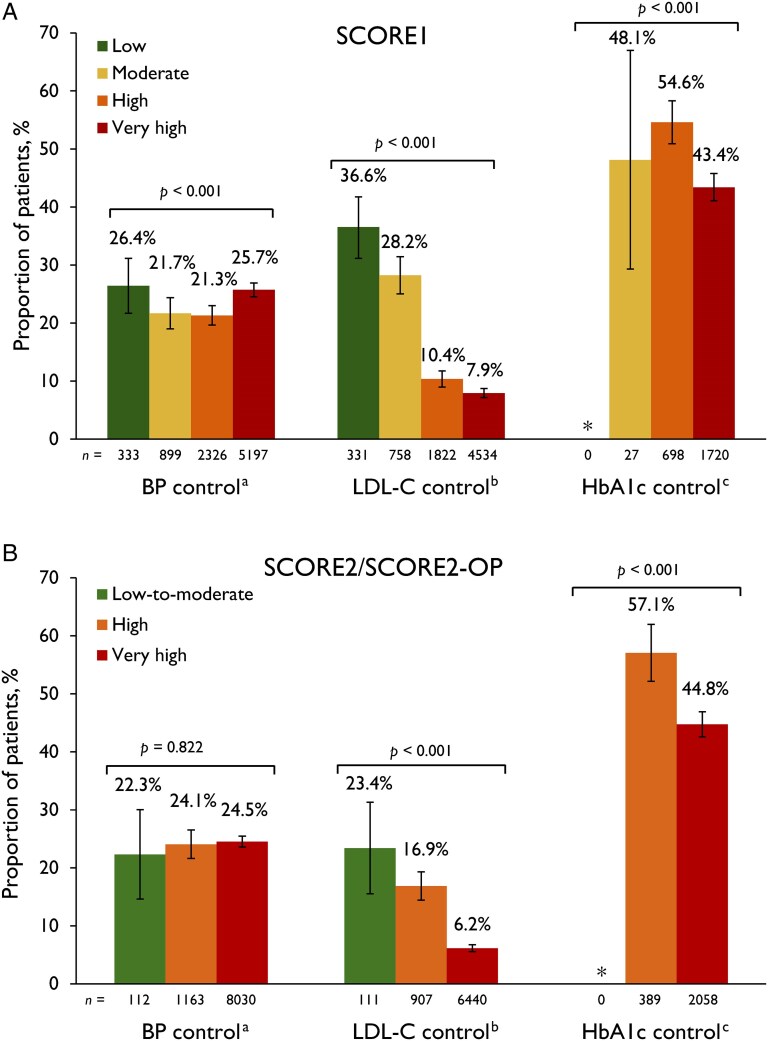
Rates of blood pressure, LDL-C, and HbA1c control stratified by calculated cardiovascular risk categories based on (*A*) SCORE1 and (*B*) SCORE2/SCORE2-OP prediction algorithms. Error bars represent 95% confidence intervals. *P*-values were estimated from *χ*^2^ tests. ^a^Blood pressure control was defined according to 2018 ESC/ESH guidelines;^20^  ^b^LDL-C control was assessed only in patients diagnosed with dyslipidaemia and defined according to SCORE1^21^ or SCORE2/SCORE2-OP^22,23^ criteria; ^c^HbA1c control was assessed only in patients diagnosed with type 2 diabetes and defined as an HbA1c < 7% (<53 mmol/mol).^24^ *Individuals with type 2 diabetes could not have low SCORE1 or low-to-moderate SCORE2/SCORE2-OP CV risk. OP, older persons.

The BP control rate varied significantly across CV risk categories when based on the SCORE1 prediction algorithm (low risk 26.4%, moderate risk 21.7%, high risk 21.3%, and very high risk 25.7%; *P* < 0.001), but not when SCORE2/SCORE2-OP criteria were used (*P* = 0.822; *Figure 3A* and *B*).

The rate of LDL-C control was significantly higher in patients with dyslipidaemia and low or moderate CV risk (36.6 and 28.2%, respectively), based on the SCORE1 prediction algorithm, compared with patients with dyslipidaemia and high or very high CV risk (10.4 and 7.9%, respectively; *P* < 0.001; *Figure 3A*). Similar results were observed for the rate of LDL-C control based on the SCORE2/SCORE2-OP prediction algorithm; the rate was significantly higher in patients with dyslipidaemia and low-to-moderate CV risk compared with those with dyslipidaemia and high or very high CV risk (23.4 vs. 16.9 and 6.2%, respectively; *P* < 0.001; *Figure 3B*).

The rate of HbA1c control was significantly higher in patients with T2D and high CV risk (54.6%) than moderate (48.2%) or very high CV risk (43.4%), based on the SCORE1 prediction algorithm (*P* < 0.001; *Figure 3A*). Similarly, when CV risk was based on the SCORE2/SCORE2-OP algorithm, HbA1c control rates were higher in patients at high CV risk than in those at very high risk (57.1 vs. 44.8%; *P* < 0.001; *Figure 3B*).

#### Physician-assessed cardiovascular risk

**Figure 4:**
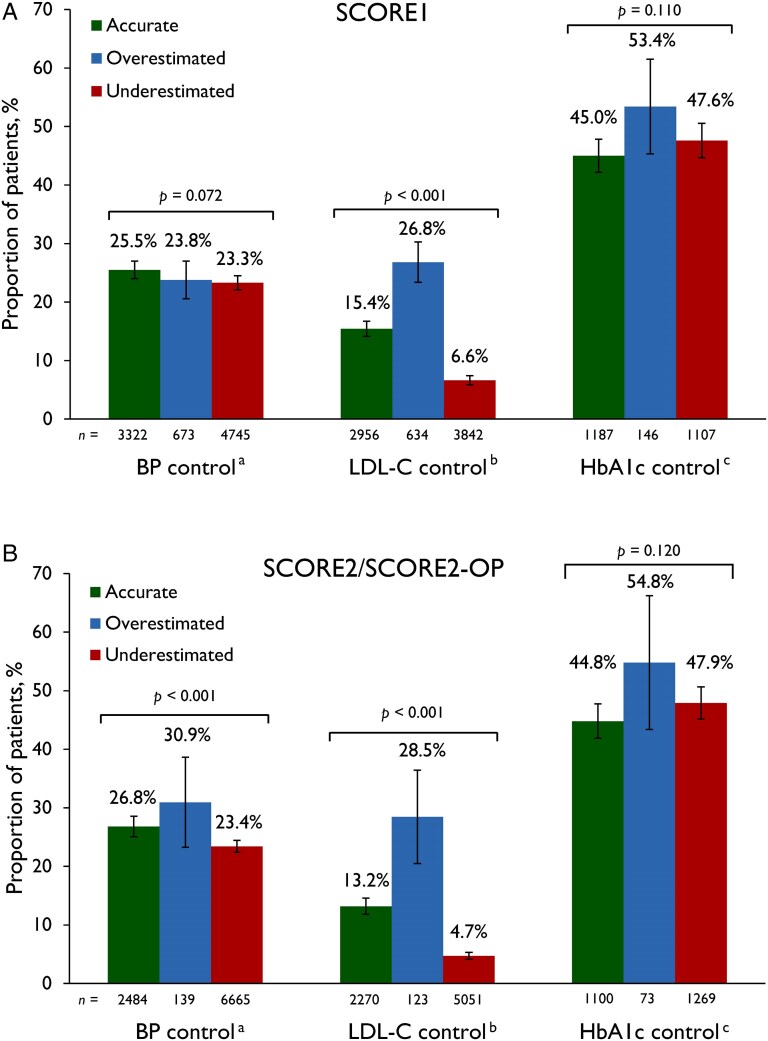
Rates of blood pressure, LDL-C, and HbA1c control stratified by the accuracy of assessment of cardiovascular risk by the physician vs. (*A*) SCORE1 and (*B*) SCORE2/SCORE2-OP prediction algorithms. Error bars represent 95% confidence intervals. *P*-values were estimated from *χ*^2^ tests. ^a^Blood pressure control was defined according to 2018 ESC/ESH guidelines^20^; ^b^LDL-C control was assessed only in patients diagnosed with dyslipidaemia and defined according to SCORE1^21^ or SCORE2/SCORE2-OP^22,23^ criteria; ^c^HbA1c control was assessed only in patients diagnosed with type 2 diabetes and defined as an HbA1c < 7% (<53 mmol/mol).^24^ OP, older persons.

The rate of BP control did not vary significantly according to the accuracy of physician-assessed CV risk vs. SCORE1 criteria (25.5% for accurately estimated CV risk vs. 23.8% for overestimated CV risk vs. 23.3% for underestimated CV risk; *P* = 0.072; *Figure 4A*). However, the rate of BP control varied according to the accuracy of physician-assessed CV risk vs. SCORE2/SCORE2-OP criteria (*P* < 0.001; *Figure 4B*). Patients with overestimated CV risk had the highest rate of BP control (30.9 vs. 26.8% for accurately estimated CV risk and 23.4% for underestimated CV risk). Patients with dyslipidaemia whose CV risk was overestimated had significantly higher rates of LDL-C control than those with accurate or underestimated CV risk vs. SCORE1 criteria (26.8 vs. 15.4 and 6.6%, respectively; *P* < 0.001), as well as vs. SCORE2/SCORE2-OP criteria (28.5 vs. 13.2 and 4.7%, respectively; *P* < 0.001). The HbA1c control rate was similar across the different levels of accuracy of CV risk assessment vs. SCORE1 (*P* = 0.110; *Figure 4A*) or SCORE2/SCORE2-OP criteria (*P* = 0.120; *Figure 4B*).

### Other subgroup analyses of control rates

#### Age, sex, and body mass index

**Figure 5:**
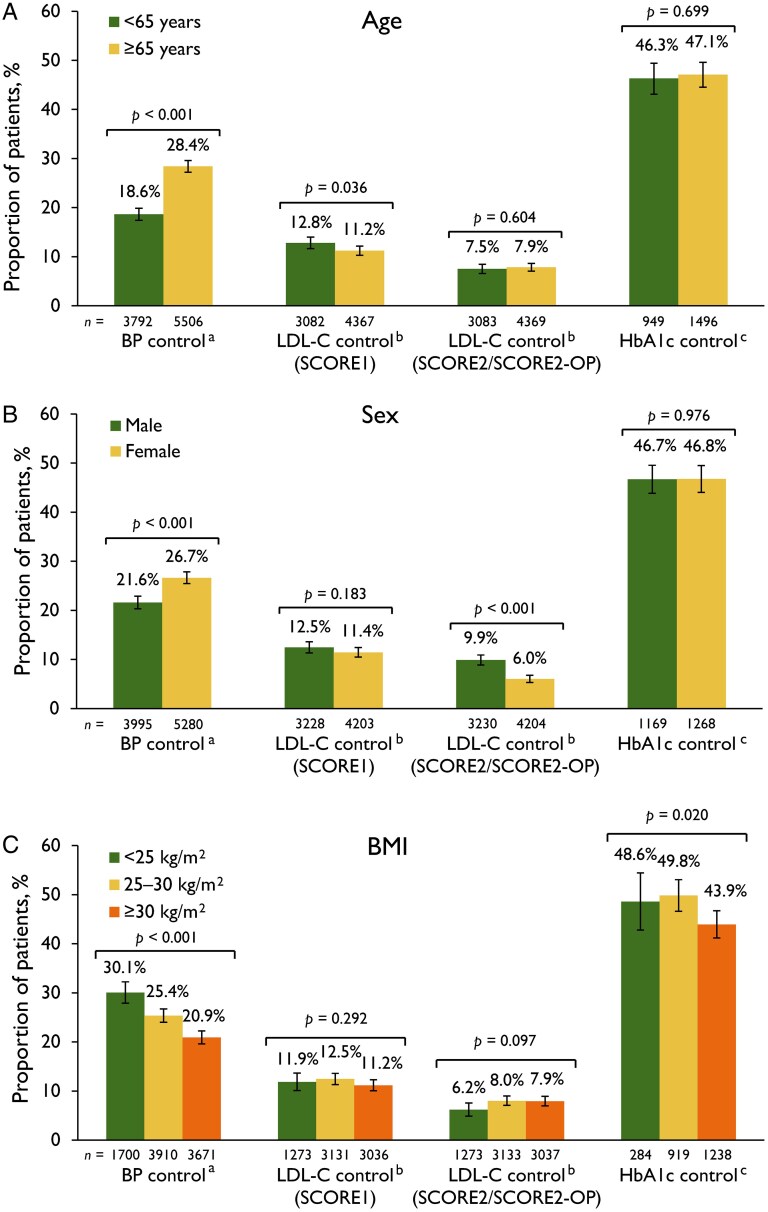
Rates of blood pressure, LDL-C, and HbA1c control stratified by (*A*) age, (*B*) sex, and (*C*) body mass index. Error bars represent 95% confidence intervals. *P*-values were estimated from *χ*^2^ tests. ^a^Blood pressure control was defined according to 2018 ESC/ESH guidelines;^20^  ^b^LDL-C control was assessed only in patients diagnosed with dyslipidaemia and defined according to SCORE1^21^ or SCORE2/SCORE2-OP^22,23^ criteria; ^c^HbA1c control was assessed only in patients diagnosed with type 2 diabetes and defined as an HbA1c < 7% (<53 mmol/mol).^24^

The rates of BP control varied significantly according to age, sex, and BMI (*Figure 5A–C*), with higher rates observed in individuals aged ≥65 years (28.4 vs. 18.6% in those aged <65 years; *P* < 0.001), in females (26.7 vs. 21.6% in males; *P* < 0.001), and in individuals with a normal BMI (30.1 vs. 25.4 or 20.9% in patients who were overweight or obese; *P* < 0.001). The LDL-C control rates according to SCORE1 or SCORE2/SCORE2-OP criteria had minor variations for age and sex, and no variations according to BMI categories (*Figure 5A–C*), with the exception of higher LDL-C control rates according to SCORE1 criteria in patients aged <65 years (12.8 vs. 11.2% in patients aged ≥65 years; *P* = 0.036; *Figure 5A*) and according to SCORE2/SCORE2-OP criteria observed in males (9.9 vs. 6.0% in females; *P* < 0.001; *Figure 5B*). The HbA1c control rates were similar for age and sex, but varied significantly depending on patient BMI (normal BMI 48.6%, overweight 49.8%, and obese 43.9%; *P* = 0.020; *Figure 5A–C*).

#### Comorbidities

**Figure 6:**
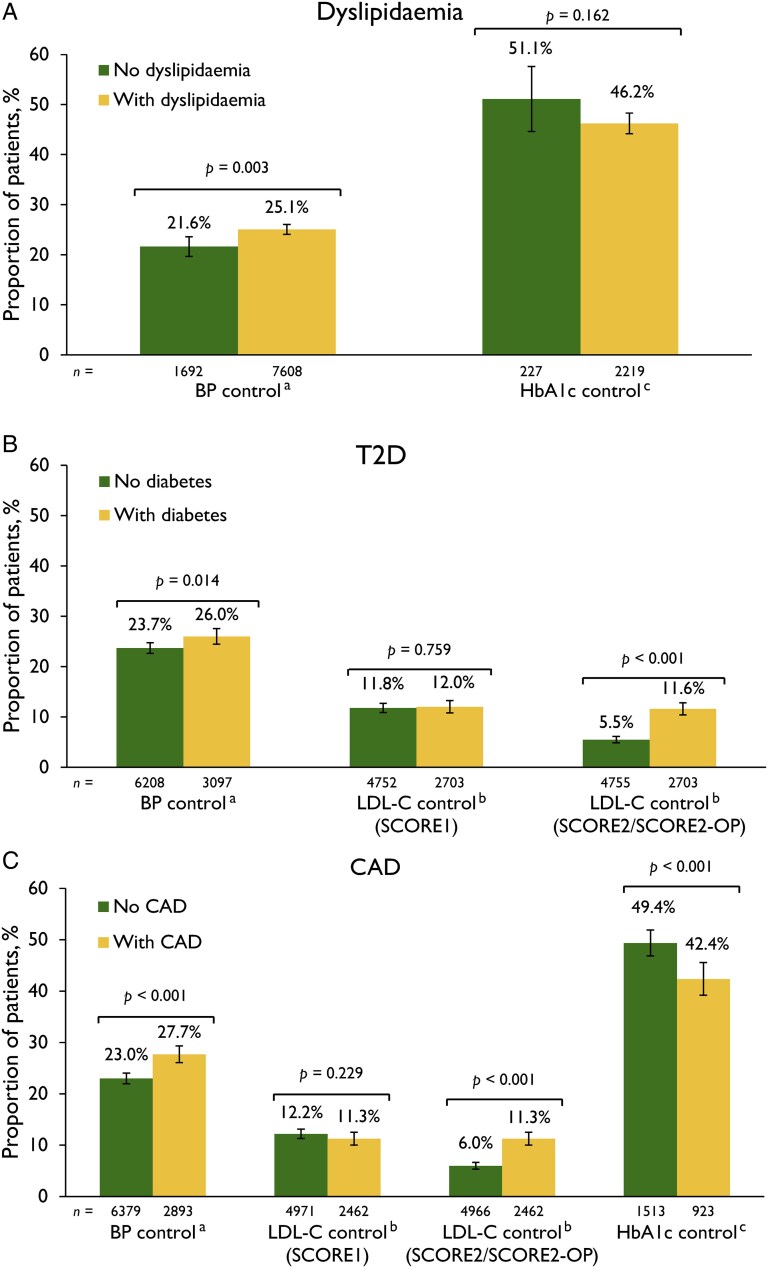
Rates of blood pressure, LDL-C, and HbA1c control stratified by the presence of (*A*) dyslipidaemia, (*B*) type 2 diabetes, and (*C*) coronary artery disease. Error bars represent 95% confidence intervals. *P*-values were estimated from *χ*^2^ tests. ^a^Blood pressure control was defined according to 2018 ESC/ESH guidelines;^20^  ^b^LDL-C control was assessed only in patients diagnosed with dyslipidaemia and defined according to SCORE1^21^ or SCORE2/SCORE2-OP^22,23^ criteria; ^c^HbA1c control was assessed only in patients diagnosed with type 2 diabetes and defined as an HbA1c < 7% (<53 mmol/mol).^24^

Patients with dyslipidaemia had higher BP control rates than patients without dyslipidaemia (*Figure 6A*). Meanwhile, patients with T2D had higher BP control rates than patients without T2D (*Figure 6B*). Similarly, patients with CAD had higher BP control rates than patients without these comorbidities (*Figure 6C*). Patients with T2D or CAD had higher LDL-C control, as assessed using SCORE2/SCORE2-OP criteria, than patients without these comorbidities (*Figure 6B* and *C*). On the contrary, patients with CAD had lower rates of HbA1c control than patients without these comorbidities (*Figure 6C*). There were no significant differences in HbA1c control rates between patients with or without dyslipidaemia (*Figure 6A*).

## Discussion

SNAPSHOT is the first large-scale observational study to evaluate control of BP, LDL-C, and HbA1c among patients with hypertension in Europe. The findings of this study provide valuable insights into CV risk assessment and rates of BP, LDL-C, and HbA1c control across Eastern and Southern European countries. These insights are especially important since there is a lack of real-world data from these countries examining control of these three CV risk factors together, and a lack of analyses in relation to the accuracy of physician CV risk assessment. Prior observational studies were generally focused on control of one CV risk factor (e.g. BP control,^25,26^ lipid targets,^27-30^ or glycaemic control^31^). This study demonstrated that most physicians underestimate CV risk and overestimate the extent of BP and LDL-C control in their patients with hypertension in routine clinical practice. Furthermore, control of BP, LDL-C, and HbA1c was suboptimal, with <25% of patients having BP control according to 2018 ESC/ESH guidelines, <12% of patients with comorbid dyslipidaemia having LDL-C control according to SCORE1 or SCORE2/SCORE2-OP criteria, and <50% of those with comorbid T2D having adequate glycaemic control [target HbA1c < 7.0% (<53 mmol/mol)].

In our study, the prevalence of additional risk factors and comorbidities was high. However, the prevalence of dyslipidaemia (81.8%), T2D (33.3%), and obesity (39.6%) was similar to that reported in the PRECISE study, which was conducted in the primary care setting and included patients with hypertension in Portugal (82.1, 32.9, and 39.1%, respectively).^32^

The physician assessment of CV risk significantly underestimated the proportion of patients with high or very high CV risk by ≥30% when compared with SCORE1 or SCORE2/SCORE2-OP prediction algorithms. Based on physician assessment, 55.7% of patients had high or very high CV risk, whereas this proportion was 85.9 and 98.8% according to guideline recommendations when using SCORE1 or SCORE2/SCORE2-OP criteria, respectively. These rates are higher than in a previous large cohort study of patients with hypertension in Spain (the PRESCAP 2006 study), which reported that 51.9% of patients had high or very high CV risk,^33^ although the PRESCAP 2006 study used the 2003 ESH/ESC guidelines to assess the CV risk.^34^ In contrast, and similar to our results, a study of hypertensive patients in Algeria, Egypt, Pakistan, Ukraine, and Venezuela, which also considered lifestyle patterns, CV risk factors, target organ damage, and associated diseases, reported that 84.8% of patients were in the high- or very high-risk category.^13^

The abovementioned assessment of CV risk was notably different between the two SCORE prediction algorithms, with a significantly higher proportion of patients identified at very high risk when SCORE2/SCORE2-OP vs. SCORE1 criteria were used. This is not surprising, since SCORE2/SCORE2-OP incorporate additional risk factors (e.g. the competing risk of non-CV mortality in SCORE2-OP) and provide a more comprehensive assessment of CV risk by estimating both fatal and non-fatal events.^9^ Other inconsistencies between analyses using SCORE1 and SCORE2/SCORE2-OP were observed in our study, mainly due to the differences in methodology between these risk assessment models. Physicians must take these differences into account when selecting the most appropriate risk prediction algorithm. Furthermore, these inconsistencies between SCORE1 and SCORE2/SCORE2-OP criteria highlight possible difficulties in the clinical practice setting of assessing CV risk according to the guidelines.

The rate of BP control in the current study was somewhat higher than estimates for Central and Eastern Europe by the Non Communicable Disease Risk Factor Collaboration (NCD-RisC),^1^ and a report by Lu *et al*.^12^ from the Czech Republic, Lithuania, Poland, and Russia. Thus, the current study estimated a BP control rate (<140/90 mmHg) of 48.2% (47.2–49.2% across countries), whereas the NCD-RisC estimated that 17% of males and 25% of females had BP <140/90 mmHg,^1^ and Lu *et al*.^12^ reported BP control rates of 30–40%. The most likely reason for this difference is that the NCD-RisC and Lu *et al.* studies collected data from the general population,^1,12^ whereas the current study included patients with hypertension being treated in routine clinical practice, and was therefore an enriched patient cohort.

The rate of LDL-C control in the current study was slightly lower than that estimated in a previous study of patients with dyslipidaemia in Spain (the TERESA study).^35^ In the current study, 11.9 and 7.7% of patients with hypertension and dyslipidaemia had LDL-C control according to SCORE1 and SCORE2/SCORE2-OP criteria, respectively, whereas in the TERESA study, the risk-based LDL-C target (according to 2019 ESC/EAS guidelines^21^) was achieved by 31.1% of patients with high or very high CV risk.^35^ The most likely reason for this difference is that the TERESA study enrolled a highly selected study population who were receiving high-intensity LLDs (i.e. atorvastatin or rosuvastatin ± ezetimibe),^35^ whereas the current study included all patients with hypertension and dyslipidaemia who were being treated in routine clinical practice.

There are limited data on HbA1c control rates among patients with hypertension and T2D in Europe. Studies tend to focus on a single patient population (either those with hypertension or those with diabetes). However, two European studies in patients with T2D also included patients taking antihypertensive treatment, which for the purposes of our discussion will be used as a proxy for indicating the presence of hypertension.^36,37^ One study found that patients with T2D who were also receiving antihypertensive treatment were less likely to meet their HbA1c targets,^37^ and in the other study, where 75.3% of patients were receiving antihypertensive treatment, the HbA1c control rate was 37.4%.^36^ A higher HbA1c control rate was observed in Japan, in the Japan Epidemiology Collaboration on Occupational Health (J-ECOH) study (44.9%),^38^ which is similar to that observed in our study (46.7%).

In the current study, patients with hypertension and dyslipidaemia and/or T2D had low rates of combined control of BP + LDL-C, BP + HbA1c, or BP + LDL-C + HbA1c. Data from one of the abovementioned European studies indicated a low combined control rate of 7.5% for patients meeting BP (<130/80 mmHg), LDL-C (<100 mg/dL), and HbA1c [<7% (<53 mmol/mol)] targets.^36^ In the J-ECOH study, 11.2% of patients had combined control of BP (<140/90 mmHg) + LDL-C (<100 mg/dL) + HbA1c [<7.0% (<53 mmol/mol)].^38^ These combined control rates for all three parameters are higher than that observed in our study (1.9%), which is most likely due to the differences between the study populations.

Our finding that physicians underestimated CV risk and overestimated BP and LDL-C control rates among their patients is consistent with previous studies.^18,39,40^ A cross-sectional study in Spanish primary care physicians found that BP control rates were higher with physician assessment than with an objective measure (46.8 vs. 11.6%).^40^ Similarly, the Supporting Hypertension Awareness and Research Europe-wide (SHARE) survey of physicians reported that the proportion of patients who failed to achieve BP targets recommended by the ESH/ESC guideline was significantly lower when assessed by general/family practitioners (42.6%) vs. cardiologists or internists (56.8 or 61.9%, respectively).^39^ With regard to LDL-C control, a previous Spanish study by Cosín-Sales *et al*.^18^ in patients with dyslipidaemia reported control rates of 62% according to the physicians’ perception, compared with 31% when using an objective measure.

Significant differences in HbA1c and LDL-C control rates were observed between CV risk groups according to SCORE1 and SCORE2/SCORE2-OP criteria. Blood pressure control rates were also significantly different across CV risk categories by SCORE1 criteria, but not by SCORE2/SCORE2-OP criteria. These findings highlight again the differences between the assessment of CV risk by the two different algorithms. Blood pressure control rates were influenced by age, sex, and BMI, whereas HbA1c control rates were only significantly affected by BMI. Among patients with hypertension, those with comorbid dyslipidaemia or T2D, as well as those with CAD, had higher BP control rates than patients who did not have these comorbidities. Meanwhile, patients with comorbid T2D, as well as patients with CAD, had higher rates of LDL-C control when SCORE2/SCORE2-OP was used. Interestingly, patients with CAD had lower rates of HbA1c control. These results suggest that patients with these CV comorbidities are receiving more optimized antihypertensive therapy and LLDs, according to guideline recommendations.^41,42^

The potential danger of overestimating BP and LDL-C control is that it may contribute to therapeutic inertia. Indeed, data from previous studies indicate that therapeutic inertia in hypertension tends to occur when BP approaches target levels,^40,43,44^ whereas physicians are more likely to intervene when patients have higher BP. In a Dutch cohort study of patients with hypertension who were managed in primary care, near-target SBP values were significantly associated with an increased likelihood of therapeutic inertia (odds ratio 1.355; 95% CI 1.090–1.684).^43^ This is most likely because treatment intensification occurs less often in patients with near-target BP, particularly those who experience adverse effects, than in patients with extremely uncontrolled BP.^43^ Therapeutic inertia in hypertension is an important contributor to poor rates of long-term BP control, especially early after initiation of antihypertensive treatment as illustrated by a Monte Carlo simulation study,^45^ and uncontrolled BP in turn increases the risk of CV and all-cause mortality.^46^ Therapeutic inertia in dyslipidaemia may also impact LDL-C control rates, as reported in the previous Spanish study by Cosín-Sales *et al*.^18^ This study reported that physicians may underestimate CV risk in patients with dyslipidaemia, thereby leading to less stringent LDL-C targets, insufficient intensification of lipid-lowering treatment, and failure to achieve LDL-C targets.^18^

Improving control of these CV risk factors is crucial, as are addressing modifiable risk factors such as an unhealthy diet, being overweight or obese, physical inactivity, and tobacco and alcohol consumption. Patients with hypertension are at a higher risk of developing ischaemic heart disease, stroke, heart failure, and chronic kidney disease, and this risk increases in the presence of comorbid dyslipidaemia and T2D.^47,48,49^ Peng *et al*.^50^ demonstrated that each additional uncontrolled risk factor was linked to a 24% rise in CV risk in hypertensive patients. Non-CV diseases should also not be overlooked; for example, a recent study showed that patients with a history of cancer were at higher risk of developing hypertension.^51^ The World Health Organization concluded in a recent global report on hypertension that increasing the percentage of people with controlled hypertension from 21% (the current control rate) to 50% would prevent a total of 76 million CV deaths globally before 2050.^47^

### Limitations

Limitations of this study include the potential for selection bias in the choice of participating physicians, and in patients who choose to participate.^52^ In future research, adjustment for confounding factors, for example, by conducting a regression analysis, may help to minimize the influence of selection bias. The cross-sectional nature of the study also precludes any conclusions about incidence, or cause and effect relationships.^52^ In addition, the guideline-defined BP and LDL-C control rates used the 2018 ESC/ESH guidelines,^20^ SCORE1,^21^ and SCORE2/SCORE2-OP algorithms,^22,23^ which have since been updated, so may not reflect control rates relative to the BP and LDL-C targets described in the 2023 ESH guidelines^7^ or the 2024 ESC guidelines.^9^ Additional, long-term, well-designed studies are needed to confirm control rates with the updated BP and LDL-C targets. Lastly, our findings may not be generalizable to patients with hypertension in other countries and/or regions.

## Conclusions

The SNAPSHOT study demonstrated that physicians from six European countries tended to overestimate control rates of BP and LDL-C, while underestimating the CV risk of their patients with hypertension during routine clinical practice. Meanwhile, the proportion of patients achieving BP, LDL-C, or HbA1c control was low, suggesting therapeutic inertia may be of concern in the real-world management of these patients. Our findings provide a real-world clinical picture of the gaps between perceived and actual CV risk among patients with hypertension, highlighting the need for interventions that will improve CV risk assessment and management in these patients. By increasing physician awareness of the need for better implementation of the ESC guideline recommendations in real-life clinical practice, we may expect that patients with hypertension will experience overall improvements in disease control and clinical outcomes.

## Supplementary Material

qcag002_Supplementary_Data

## Data Availability

The datasets used and analysed during the current study are available from the corresponding author upon reasonable request.
