# Supplementary material for: Cardiovascular risk assessment and risk factor control in patients with hypertension in Europe: the SNAPSHOT study
Source: Eur Heart J Qual Care Clin Outcomes. 2026 Jan 19;12(4):498–512. doi: 10.1093/ehjqcco/qcag002 (PMC13288740; doi:10.1093/ehjqcco/qcag002)
Supplement: qcag002_Supplementary_Data [file qcag002_supplementary_data.pdf]

Cardiovascular Risk Assessment and Risk Factor Control in Patients with Hypertension in Europe: the SNAPSHOT Study

Dragos Vinereanu, Miguel Camafort, Anastase Dzudie, Branislava Ivanovic, Jose Maria Mostaza, Ivan Pecin, Arman Postadzhiyan, Tamaz Shaburishvili, Julien Magne

Supplementary materials

Supplementary Table S1 Clinical centre types of participating physicians

Clinical centre	Participating physicians (N = 513)[a]
Specialty of physician, *n* (%)	
General practitioner	357 (69.6)
Cardiology	89 (17.4)
Endocrinology	32 (6.2)
Internal medicine	32 (6.2)
Neurology	3 (0.6)
Institute type, *n* (%)	
Outpatient clinic	341 (66.5)
Hospital	90 (17.5)
Primary care facility	82 (16.0)
Location, *n* (%)	
Urban	441 (86.0)
Rural	72 (14.0)

[a]Each clinical centre may have had more than one participating physician.

Supplementary Table S2 Systematic COronary Risk Evaluation chart 1 (SCORE1) cardiovascular risk (CV) categories, provided in the 2016 European Guidelines on cardiovascular disease prevention in clinical practice and revised in the 2019 European Society of Cardiology (ESC)/European Atherosclerosis Society guidelines[1,2]

CV risk category[a]	Criteria
Low	• SCORE1 0–2%
Moderate	• SCORE1 2–5%<br>• Patients aged <50 years with T2D without target organ damage and time since diagnosis <10 years<br>• Grade 2 hypertension (SBP 160–179 mmHg and/or DBP 100–109 mmHg)
High	• SCORE1 5–10%<br>• Patients aged ≥50 years with T2D without target organ damage and time since diagnosis <10 years<br>• T1D without target organ damage<br>• T2D with time since diagnosis ≥10 years<br>• TC >310 mg/dL<br>• LDL-C >190 mg/dL<br>• Grade 3 hypertension (SBP ≥180 mmHg and/or DBP ≥110 mmHg)<br>• Hypertensive LV hypertrophy<br>• Moderate CKD
Very high	• SCORE1 ≥10%<br>• Severe CKD<br>• DM with target organ damage<br>• Patient with a family history of CVD<br>• Presence of at least one additional comorbidity (ACS, MI, angina, stroke, TIA, coronary or other arterial revascularisation, or PAD)

[a]If a patient has items from different levels of score, the highest level was used.

ACS, acute coronary syndrome; CKD, chronic kidney disease; CVD, cardiovascular disease; DM, diabetes mellitus; LV, left ventricular; MI, myocardial infarction; PAD, peripheral artery disease; T1D, type 1 diabetes; T2D, type 2 diabetes; TC, total cholesterol; TIA, transient ischaemic attack.

Supplementary Table S3 Guideline recommendations and Systematic COronary Risk Evaluation chart 2 (SCORE2; for adults aged 40–69 years) and SCORE2-older persons (SCORE2-OP; for adults aged ≥70 years) CV risk categories[3,4]

**CV risk category**[a]	**Criteria**
Low-to-moderate	• Age <50 years: SCORE2 <2.5%<br>• Age 50–69 years: SCORE2 <5%<br>• Age ≥70 years: SCORE2-OP <7.5%<br>• Patients with T2D without target organ damage and time since diagnosis <10 years and without additional CV risk factors[b]
High	• Moderate CKD<br>• Patients with T1D without target organ damage<br>• Patients with T2D, without target organ damage, time since diagnosis <10 years and with additional CV risk factors<br>• Patients with T2D without target organ damage and time since diagnosis ≥10 years<br>• Age <50 years: SCORE2 2.5–7.5%<br>• Age 50–69 years: SCORE2 5–10%<br>• Age ≥70 years: SCORE2-OP 7.5–15%
Very high	• Severe CKD<br>• DM with target organ damage<br>• DM with microvascular complications<br>• Presence of at least one additional comorbidity (ACS, MI, angina, stroke, TIA, coronary or other arterial revascularisation, or PAD)<br>• Age <50 years: SCORE2 ≥7.5%<br>• Age 50–69 years: SCORE2 ≥10%<br>• Age ≥70 years: SCORE-OP ≥15%

[a]If a patient has items from different levels of score, the highest level was used.

[b]By definition of the population, all patients have hypertension, so they have at least one CV risk factor.

Supplementary Table S4 Algorithm for determining blood pressure (BP) control, according to the 2018 ESC/European Society of Hypertension (ESH) guidelines[5]

**Step**	**BP control rate definition**
1st step	Patients with SBP <140 mmHg and DBP <90 mmHg
2nd step	In non-treated patients (not under antihypertensive medication):<br>• For patients aged <80 years, BP control is defined if BP <140/90 mmHg<br>• For patients aged ≥80 years, BP control is defined as BP <160/90 mmHg<br>In treated patients:<br>• For patients aged <65 years without CKD, BP control is defined as SBP ≤130 mmHg and DBP <80 mmHg<br>• For patients aged <65 years with CKD and patients aged ≥65 years, BP control is defined as BP <140/80 mmHg

DBP, diastolic blood pressure; SBP, systolic blood pressure

Supplementary Table S5 Algorithm for determining low-density lipoprotein cholesterol (LDL-C) control, according to SCORE1 and SCORE2/SCORE2-OP criteria[1,3,4]

**CV risk assessment**[a]	**LDL-C control definition**
SCORE1	• For patients with very high CV risk (calculated), LDL-C control is defined as LDL-C <1.4 mmol/L (<55 mg/dL)<br>• For patients with high CV risk (calculated), LDL-C control is defined as LDL-C <1.8 mmol/L (<70 mg/dL)<br>• For patients with moderate CV risk (calculated), LDL-C control is defined as LDL-C <2.6 mmol/L (<100 mg/dL)<br>• For patients with low CV risk (calculated), LDL-C control is defined as LDL-C <3 mmol/L (<116 mg/dL)
SCORE2/SCORE2-OP	• For patients with very high CV risk (calculated), LDL-C control is defined as LDL-C <1.4 mmol/L (<55 mg/dL)<br>• For patients with high CV risk (calculated), LDL-C control is defined as LDL-C <1.8 mmol/L (<70 mg/dL)<br>• For patients with low-to-moderate CV risk (calculated), dyslipidaemia control is defined as LDL-C <2.6 mmol/L (<100 mg/dL)

[a]The choice of CV risk calculated (SCORE1, SCORE2/2-OP, or both) was country specific.

OP, older persons.

Supplementary Table S6 Cardiovascular risk categories and BP, LDL-C and glycated haemoglobin (HbA1c) control rates by specific country

	**Bulgaria (*N* = 3260)**	**Croatia (*N* = 1296)**	**Georgia (*N* = 585)**	**Romania (*N* = 2522)**	**Serbia (*N* = 1180)**	**Spain (*N* = 464)**
CV risk category, *n* (%)						
Physician assessment	*n* = 3257	*n* = 1296	*n* = 584	*n* = 2509	*n* = 1180	*n* = 464
Low	403 (12.4)	103 (8.0)	111 (19.0)	124 (4.9)	107 (9.1)	33 (7.1)
Moderate	1403 (43.1)	394 (30.4)	182 (31.2)	620 (24.7)	496 (42.0)	137 (29.5)
High	1049 (32.2)	391 (30.2)	164 (28.1)	798 (31.8)	446 (37.8)	161 (34.7)
Very high	402 (12.3)	408 (31.5)	127 (21.8)	967 (38.5)	131 (11.1)	133 (28.7)
SCORE1	*n* = 2767	*n* = 1296	*n* = 536	*n* = 2521	*n* = 1176	*n* = 461
Low	42 (1.5)	57 (4.4)	12 (2.2)	24 (1.0)	146 (12.4)	53 (11.5)
Moderate	268 (9.7)	229 (17.7)	20 (3.7)	111 (4.4)	196 (16.7)	75 (16.3)
High	808 (29.2)	475 (36.7)	233 (43.5)	333 (13.2)	340 (28.9)	137 (29.7)
Very high	1649 (59.6)	535 (41.3)	271 (50.6)	2053 (81.3)	494 (42.0)	196 (42.5)
SCORE2/2-OP	*n* = 3260	*n* = 1296	*n* = 585	*n* = 2522	*n* = 1180	*n* = 464
Low-to-moderate	0	59 (4.6)	2 (0.3)	0	0	51 (11.0)
High	285 (8.7)	321 (24.8)	89 (15.2)	125 (5.0)	175 (14.8)	168 (36.2)
Very high	2975 (91.3)	916 (70.7)	494 (84.4)	2397 (95.0)	1005 (85.2)	245 (52.8)
Accuracy of CV risk assessment by physicians, *n* (%), relative to:						
SCORE1	*n* = 2765	*n* = 1296	*n* = 535	*n* = 2509	*n* = 1176	*n* = 461
Accurate	780 (28.2)	624 (48.2)	165 (30.8)	1084 (43.2)	418 (35.5)	251 (54.5)
Overestimated	98 (3.5)	200 (15.4)	25 (4.7)	118 (4.7)	165 (14.0)	68 (14.8)
Underestimated	1887 (68.3)	472 (36.4)	345 (64.5)	1307 (52.1)	593 (50.4)	142 (30.8)
SCORE2/SCORE2-OP	*n* = 3257	*n* = 1296	*n* = 584	*n* = 2509	*n* = 1180	*n* = 464
Accurate	467 (14.3)	486 (37.5)	135 (23.1)	1003 (40.0)	157 (13.3)	236 (50.9)
Overestimated	8 (0.3)	68 (5.3)	10 (1.7)	20 (0.8)	9 (0.8)	24 (5.2)
Underestimated	2782 (85.4)	742 (57.3)	439 (75.2)	1486 (59.2)	1014 (85.9)	204 (44.0)

BP control rate, *n*/*N*[a] (%) [95% CI]						
Physician assessment	2497/3239 (77.1) [75.6–78.5]	1021/1284 (79.5) [77.3–81.7]	400/553 (72.3) [68.6–76.1]	1768/2471 (71.6) [69.8–73.3]	873/1165 (74.9) [72.5–77.4]	304/448 (67.9) [63.5–72.2]
BP <140/90 mmHg	1277/3260 (39.2) [37.5–40.9]	750/1296 (57.9) [55.2–60.6]	274/585 (46.8) [42.8–50.9]	1260/2522 (50.0) [48.0–51.9]	679/1180 (57.5) [54.7–60.4]	246/464 (53.0) [48.5–57.6]
2018 ESC/ESH guidelines[b]	749/3259 (23.0) [21.5–24.4]	310/1296 (23.9) [21.6–26.2]	113/585 (19.3) [16.1–22.5]	719/2521 (28.5) [26.8–30.3]	244/1180 (20.7) [18.4–23.0]	140/464 (30.2) [26.0–34.4]
LDL-C control rate, *n*/*N*[a] (%) [95% CI]						
Physician assessment	964/1590 (60.6) [58.2–63.0]	558/1030 (54.2) [51.1–57.2]	159/228 (69.7) [63.8–75.7]	1166/2349 (49.6) [47.6–51.7]	528/1096 (48.2) [45.2–51.1]	264/413 (63.9) [59.3–68.6]
SCORE1[c]	168/1834 (9.2) [7.8–10.5]	179/1267 (14.1) [12.2–16.1]	50/254 (19.7) [14.8–24.6]	221/2489 (8.9) [7.8–10.0]	120/1168 (10.3) [8.5–12.0]	147/443 (33.2) [28.8–37.6]
SCORE2/SCORE2-OP[d]	104/1781 (5.7) [4.6–6.7]	117/1193 (9.2) [7.6–10.8]	44/254 (17.3) [12.7–22.0]	160/2490 (6.4) [5.5–7.4]	37/1168 (3.2) [2.2–4.2]	113/443 (25.5) [21.5–29.6]
HbA1c control rate, *n*/*N*[a] (%) [95% CI]						
HbA1c <7% (<53 mmol/mol)[e]	349/743 (47.0) [43.4–50.6]	170/363 (46.8) [41.7–52.0]	33/70 (47.1) [35.4–58.8]	408/894 (45.6) [42.4–48.9]	107/229 (46.7) [40.3–53.2]	76/148 (51.4) [43.3–59.4]

[a]Patients with missing data were considered as having 'inconclusive' control and were excluded from the denominator value when calculating the control rate. The combination of two or more factors was only considered 'controlled' when all factors were controlled; if one or two factors of a combination were 'controlled' but the second or third factor was missing (or 'inconclusive'), these patients were excluded from the analysis.

[b]2018 ESC/ESH guidelines: in non-treated patients (not under antihypertensive medication): (1) for patients aged <80 years, BP control is defined as BP <140/90 mmHg; (2) for patients aged ≥80 years, BP control is defined as BP <160/90 mmHg. In treated patients: (1) for patients aged <65 years without CKD, BP control is defined as SBP ≤130 mmHg and DBP <80 mmHg; (2) for patients aged <65 years with CKD and patients aged ≥65 years, BP control is defined as BP <140/80 mmHg.[2,5]

[c]SCORE1: For patients with very high CV risk (calculated), LDL-C control is defined as LDL-C <1.4 mmol/L (<55 mg/dL). For patients with high CV risk (calculated), LDL-C control is defined as LDL-C <1.8 mmol/L (<70 mg/dL). For patients with moderate CV risk (calculated), LDL-C control is defined as LDL-C <2.6 mmol/L (<100 mg/dL). For patients with low CV risk (calculated), LDL-C control is defined as LDL-C <3 mmol/L (<116 mg/dL).[1]

[d]SCORE2/SCORE2-OP: For patients with very high CV risk (calculated), LDL-C control is defined as LDL-C <1.4 mmol/L (<55 mg/dL). For patients with high CV risk (calculated), LDL-C control is defined as LDL-C <1.8 mmol/L (<70 mg/dL). For patients with low-to-moderate CV risk (calculated), LDL-C control is defined as LDL-C <2.6 mmol/L (<100 mg/dL).[3,4]

[e]HbA1c control rates were evaluated per the ADA 2020 glycaemic targets.[6]

ADA, American Diabetes Association.

References

1. Piepoli MF, Hoes AW, Agewall S, Albus C, Brotons C, Catapano AL, et al. 2016 European Guidelines on cardiovascular disease prevention in clinical practice. The Sixth Joint Task Force of the European Society of Cardiology and Other Societies on Cardiovascular Disease Prevention in Clinical Practice (constituted by representatives of 10 societies and by invited experts). Developed with the special contribution of the European Association for Cardiovascular Prevention & Rehabilitation (EACPR). *Eur J Prev Cardiol* 2016;**23**:NP1–NP96. https://doi.org/10.1177/2047487316653709

2. Mach F, Baigent C, Catapano AL, Koskinas KC, Casula M, Badimon L, et al. 2019 ESC/EAS Guidelines for the management of dyslipidaemias: lipid modification to reduce cardiovascular risk. *Eur Heart J* 2020;**41**:111–188. https://doi.org/10.1093/eurheartj/ehz455

3. SCORE2 Working Group, ESC Cardiovascular Risk Collaboration. SCORE2 risk prediction algorithms: new models to estimate 10-year risk of cardiovascular disease in Europe. *Eur Heart J* 2021;**42**:2439–2454. https://doi.org/10.1093/eurheartj/ehab309

4. SCORE2-OP Working Group, ESC Cardiovascular Risk Collaboration. SCORE2-OP risk prediction algorithms: estimating incident cardiovascular event risk in older persons in four geographical risk regions. *Eur Heart J* 2021;**42**:2455–2467. https://doi.org/10.1093/eurheartj/ehab312

5. Williams B, Mancia G, Spiering W, Agabiti Rosei E, Azizi M, Burnier M, et al. 2018 ESC/ESH Guidelines for the management of arterial hypertension: the Task Force for the management of arterial hypertension of the European Society of Cardiology and the European Society of Hypertension. *J Hypertens* 2018;**36**:1953–2041.

https://doi.org/10.1097/HJH.0000000000001940

6. American Diabetes Association. 6. Glycemic Targets: Standards of Medical Care in Diabetes—2020. *Diabetes Care* 2019;**43**:S66–S76. https://doi.org/10.2337/dc20-S006
